# Single-cell RNA-seq study determines the ontogeny of macrophages in glioblastomas

**DOI:** 10.1186/s13059-017-1375-z

**Published:** 2017-12-21

**Authors:** Chenghang (Chuck) Zong

**Affiliations:** 1 Department of Molecular and Human Genetics, Baylor College of Medicine, One Baylor Plaza, Houston, TX 77030 USA; 2 Integrative Molecular and Biomedical Sciences Graduate Program, Baylor College of Medicine, One Baylor Plaza, Houston, TX 77030 USA; 3 Dan L Duncan Comprehensive Cancer Center, Baylor College of Medicine, One Baylor Plaza, Houston, TX 77030 USA; 4 McNair Medical Institute, Baylor College of Medicine, One Baylor Plaza, Houston, TX 77030 USA

## Abstract

A large-scale single-cell RNA-seq analysis of tumor-associated macrophages in gliomas has unveiled a new aspect of the complex tumor microenvironment and new biomarkers.

## Introduction

Tumor heterogeneity is determined by both heterogeneous tumor cells and a complex tumor microenvironment. Profiling of different types of cells from among the complex cell population in the tumor microenvironment, as well as determining their lineage and their phenotypes, is critical for researchers to understand how such cells interact with tumor cells. The rapid development of large-scale single-cell RNA-seq platforms has supported recent endeavors in profiling the complex immune system in both normal and tumor samples, which have begun to unveil different proportions and subtypes of immune cells systematically [1–7]. The article by Müller et al. [8] in this issue of *Genome Biology* presents another contribution using single-cell RNA-seq, this time focusing on macrophages in adult glioblastomas. Glioblastoma is the most common and most aggressive from of primary adult brain tumor [9]; therefore, characterizing the heterogeneous population of macrophages in glioblastomas is necessary to provide better understanding of this aggressive tumor, as well as to design more effective therapeutics to treat it.

## The binary ontogeny model versus the continuum model of macrophages

Tumor-associated macrophages (TAMs) are abundant in gliomas and evidence suggests that these TAMs contribute to therapy resistance. In a previous study focusing on two classes of gliomas (astrocytoma and oligodendroglioma), Venteicher et al. [6] identified a continuum model of macrophages crossing from a microglia-like state to a macrophage-like state, and they noticed that the degree of transition state is unique to different patients. In contrast to this observation, Müller at al. identified a clear binary model across the patient samples, clearly separating the two types of resident macrophage populations (Fig. 3a in [8]). This distinct pattern indicates different underlying mechanisms in low-grade glioma and glioblastoma. The binary model directly substantiates different cell ontogenies, whereas the continuum model suggests the potential dynamic transition between different states of macrophage. There is a long-running debate about the extent to which macrophages from peripheral blood adopt the phenotype of tissue-resident phagocytes in malignant conditions, but both studies provide evidence that different proportions of macrophages vary with different grades and subtypes of tumors. Future investigations are greatly needed so that researchers can discover why there are continuous transitions in low-grade gliomas, but significantly fewer or none in glioblastomas.

Blood-derived macrophages have much higher infiltration rates in glioblastomas than in low-grade gliomas. Compared to microglia-derived TAMs, blood-derived TAMs upregulate immunosuppressive cytokines, markers of active phagocytosis, and markers of an activated tricarboxylic (TCA) cycle. Therefore, blood-derived TAMs play important roles in immunosuppression. Müller et al. showed that the evaluated activities of blood-derived-TAMs, but not of microglial-TAMs, significantly correlate with inferior overall survival in grade II–III low-grade glioma (Fig. 5b in [8]). With different lineages of macrophage being associated with different roles in tumors, the therapeutic approaches that target both microglia-derived TAMs and blood-derived TAMs indiscriminately are called into question and could be replaced by therapies that only target blood-derived TAMs.

For future clinical applications, Müller et al. [8] discovered 66 new gene sets that can be used as biomarkers to distinguish the different lineages of the macrophage cell subsets. Besides the known P2RY12, the authors further validated CD49D and HLA-DRA from their gene sets as markers of macrophage populations.

## Dependence of TAMs on a subtype of tumors

In addition to a distinct pattern of TAMs of different ontogeny, it is interesting to characterize whether there is a pattern of heterogeneity in the tumor microenvironment that is associated with the tumors’ subtype or molecular features. Indeed, Müller et al. identified different proportions of blood-derived TAMs versus microglial-derived macrophages in oligodendrogliomas and astrocytomas, as well as in glioblastomas of classic, mesenchymal or proneural subtypes. The data show a significant increase in blood-derived TAMs, but not in microglial TAMs, in glioblastoma compared to low-grade glioma (Fig. 5a in [8]). The mesenchymal subtype shows an even higher degree of enrichment for blood-derived TAMs. In addition, astrocytomas have a degree of microglia infiltration that is significantly higher than that in oligodendrogliomas and glioblastomas. These observations strongly support the association between the heterogeneity of the TAMs and the subtype of the tumor. Therapies that include normalizing the blood–brain barrier will potentially have different outcomes due to the different subtypes of gliomas or glioblastomas that occur in patients.

## Noncanonical macrophage activation in glioblastoma

The heterogeneity of the tumor microenvironment is reflected not only in the complex composition of the different cell types but also by possible abnormal phenotypes. Indeed, Müller et al. [8] identified an unexpected noncanonical activation with co-expression of both the M2 subtype of macrophage marker IL10 and the M1 subtype marker tumor necrosis factor α (TNF-α). The authors further confirmed this observation at the protein level. This noncanonical activation occurs in both microglia and blood-derived macrophages, indicating that mixed signaling between tumor cells and their microenvironment leads to abnormal simultaneous M1 and M2 activation. Interestingly, this simultaneous M1–M2 expression is also observed in a recent study of traumatic brain injury [10], indicating that this noncanonical macrophage activation might occur more widely, although the mechanisms underlying it are still unclear.

## Using single-cell RNA-seq to study TAMs

A major advance utilized in this study is the use of single-cell RNA-seq to look in detail at the two different macrophage populations. It is also worth mentioning that, in this study, the authors conducted the single-cell RNA-seq across different platforms, using Fluidigm (SMARTer and SMART-seq2 chemistry) as well as 10x Drop-seq. Interestingly, the sensitivities of the two platforms were different, with better sensitivity supported by the Fluidigm platform. The two populations can be distinguished at the axis of principle component 1 in the principle component analysis. Furthermore, the 10x genomics data did not distinguish the two lineages as clearly as did the Fluidigm data. The 10x genomics data indicate that although 10x genomics data can sample a very large number of cells and profile different types of cells, the limited sensitivity provided by this platform may reduce its ability to identify cell subtypes, which should also be borne in mind for future studies.

## References

[1] Gaublomme JT, Yosef N, Lee Y, Gertner RS, Yang LV, Wu C (2015). Single-cell genomics unveils critical regulators of Th17 cell pathogenicity. Cell.

[2] Bjorklund AK, Forkel M, Picelli S, Konya V, Theorell J, Friberg D (2016). The heterogeneity of human CD127(+) innate lymphoid cells revealed by single-cell RNA sequencing. Nat Immunol.

[3] De Simone M, Arrigoni A, Rossetti G, Gruarin P, Ranzani V, Politano C (2016). Transcriptional landscape of human tissue lymphocytes unveils uniqueness of tumor-infiltrating T regulatory cells. Immunity.

[4] Plitas G, Konopacki C, Wu K, Bos PD, Morrow M, Putintseva EV (2016). Regulatory T cells exhibit distinct features in human breast cancer. Immunity.

[5] Tirosh I, Izar B, Prakadan SM, Wadsworth MH, Treacy D, Trombetta JJ (2016). Dissecting the multicellular ecosystem of metastatic melanoma by single-cell RNA-seq. Science.

[6] Venteicher AS, Tirosh I, Hebert C, Yizhak K, Neftel C, Filbin MG, et al. Decoupling genetics, lineages, and microenvironment in IDH-mutant gliomas by single-cell RNA-seq. Science. 2017;355(6332). doi: 10.1126/science.aai8478. 10.1126/science.aai8478 PMC5519096 28360267

[7] Zheng C, Zheng L, Yoo JK, Guo H, Zhang Y, Guo X (2017). Landscape of infiltrating T cells in liver cancer revealed by single-cell sequencing. Cell.

[8] Müller SGK, Liu SJ, Alvorado B, Carrera D, Bhaduri A, Watchmaker P et al. Single-cell profiling of human gliomas reveals 1 macrophage ontogeny as a basis for regional differences in macrophage activation in the tumor microenvironment. Genome Biol. 2017. doi:10.1186/s13059-017-1362-4. 10.1186/s13059-017-1362-4 PMC5738907 29262845

[9] Ohgaki H, Kleihues P (2005). Population-based studies on incidence, survival rates, and genetic alterations in astrocytic and oligodendroglial gliomas. J Neuropathol Exp Neurol.

[10] Morganti JM, Riparip LK, Rosi S (2016). Call off the dog(ma): M1/M2 polarization is concurrent followingtraumatic brain injury. PLoS One.

